# Editorial: Facing cancer together: current research and future perspectives on psychosocial, relational, and intervention approaches for couples

**DOI:** 10.3389/fpsyg.2023.1238868

**Published:** 2023-07-14

**Authors:** Linda Charvoz, Chiara Acquati, Aurélie Untas, Tanja Zimmermann

**Affiliations:** ^1^Faculty of Social Work (HETSL | HES-SO), University of Applied Sciences and Arts of Western Switzerland, Lausanne, Switzerland; ^2^Graduate College of Social Work, University of Houston, Houston, TX, United States; ^3^Department of Clinical Sciences, Tilman Fertitta Family College of Medicine, University of Houston, Houston, TX, United States; ^4^Department of Health Disparities Research, The University of Texas MD Anderson Cancer Center, Houston, TX, United States; ^5^Laboratoire de Psychopathologie et Processus de Santé, Université Paris Cité, Boulogne Billancourt, France; ^6^Department of Psychosomatics and Psychotherapy, Hannover Medical School, Hanover, Germany

**Keywords:** cancer, couples, relational factors, dyadic processes, individual and dyadic outcomes, prevention and intervention programs, dyadic measurement and research methods

## Theoretical background

An extensive number of studies has demonstrated that patients with cancer as well as intimate partners experience significant rates of psychological distress and that both need to be supported adjusting to the multiple types of burden associated with the disease (Kaye and Gracely, 1993; Heckel et al., 2015). Since then, cancer-related stress and coping have been regarded as interdependent processes (Bodenmann, 1997; Revenson et al., 2005). Cancer has been described as a “we-disease” (Kayser et al., 2007; Leuchtmann and Bodenmann, 2017) and couples coping with the illness has been conceptualized and investigated through several models, such as the Relationship-Focused Coping Model (Delongis and O'Brien, 1990; Coyne and Smith, 1991), the Systemic-Transactional Model (Bodenmann, 1997), the Communal Coping Model (Lyons et al., 1998), the Relational-Cultural Model (Kayser et al., 2007; Kayser and Acquati, 2019) and the Developmental-Contextual Coping Model (Berg and Upchurch, 2007). On the basis of these different models, programs reducing psychological distress and enhancing dyadic processes were developed (e.g., Kayser and Scott, 2008; Badr et al., 2015; Zimmermann, 2015).

Recent works have explored couples coping with cancer integrating different variables. For examples, studies displayed that various relational factors (e.g., attachment style, mutuality, etc.) and different close relationship processes (e.g., dyadic coping, communication, shared-decision making, etc.) have an impact on individual (e.g., physical and psychological health, quality of life) and dyadic (e.g., marital quality and satisfaction, sexual and reproductive health, etc.) outcomes (Kayser and Acquati, 2019; Meier et al., 2019; Bodschwinna et al., 2021).

## This special Research Topic

Despite growing awareness and recognition of the psychosocial impact of cancer on close relationships, several gaps were identified in the extant literature. The present Research Topic was therefore aimed at addressing current aspects of limitations and to inform future directions.

The impact of certain relational factors and dyadic processes (e.g., authenticity, self-disclosure, etc.) on the quality of life and wellbeing of the patients, partners and couples remain to be determined. Furthermore, studies are needed to investigate the mechanisms (i.e., mediators and/or moderators) that regulate the associations between relational factors and/or dyadic processes affecting individual and/or dyadic outcomes. The modest effects reported by the prevention programs and/or clinical interventions developed to date suggest that more studies are needed to better understand for whom (e.g., which type of patients or of couples? which type of cancers?) and when these programs are beneficial. Additionally, factors associated with positive results, timing of the intervention, and the mechanism for therapeutic change should be considered. Many studies have focused on certain types of cancer (e.g., breast, lung, or prostate cancer) and couples (e.g., heterosexual couples, couples from elevated socioeconomic backgrounds). The aim of this issue is also to highlight studies conducted on different types of cancer, stages of the disease, and groups currently understudied and underserved. Additional studies are also needed to explore the experiences of patients and partners across the lifespan and the cancer care continuum.

This current Research Topic contains original articles and systematic reviews. It examines the psychosocial experience of couples facing cancer with the goal to highlight innovative methods and approaches, whether quantitative, qualitative, or a mixed-methods. This Research Topic begins with a systematic review, in which Fugmann et al. investigated the ***impact of cancer on marital dissolution***. The authors collected empirical evidence on the research questions whether a cancer diagnosis in general or the type of cancer affects the divorce rate. In addition, the methodological biases of the studies included in the review were discussed.

Three notable themes emerge throughout the 10 other contributions of this topic. One **first** central theme is the exploration of the *relationships between individual factors, close relationship processes, and individual and dyadic outcomes*. Through their qualitative study, Bodschwinna et al. developed a subtle understanding of the different types of coping (individual coping, dyadic coping and social support) used by couples facing hematological cancer. While the results reported differences between patients and partners with regard to coping and social support strategies, all of these results agreed that the different strategies were mainly focused on the wellbeing of the patient. Brosseau et al. explored through focus groups the individual and the close relationship factors obstructing and facilitating cancer-related dyadic efficacy, a predictor of positive individual and relational outcomes. Four main categories of influence could be highlighted including fluid facilitators and obstacles with respect to time and domain. The study of Lyons et al. investigated the potential moderating roles of two socio-demographic variables (age et sex) on the link between close relationship processes (active engagement and protective buffering) and depression in couples facing cancer. Their results confirmed the importance of the role of the close relationship processes on the level of depression reported by each of the partners, but also the importance of the role of the couples' sex and age. On the basis of individual (coping with cancer, body image) and relational (dyadic coping, relational closeness) factors, Saita et al. identified different dyadic profiles in couples facing breast cancer. These authors highlighted the differences in functioning between couples, with functional relationships (= both partners are coherent manner in terms of coping and facing cancer) reported lower rates of depression and anxiety.

A second theme developed in this topic is the ***sexual and intimacy adjustment*** in couples facing cancer. The purpose of the Stulz et al. study was to examine whether the congruence of dyadic coping within couples with a colon cancer improves emotional and sexual adjustment. In a longitudinal study, Rottmann et al. examined whether patient- and partner- characteristics (demographic and health characteristics, quality of life factors, cancer treatment) as well as relationship-related characteristics (emotional closeness, dyadic coping) were associated with sexual activity of couples facing breast cancer. Reese et al. explored the experiences of couples facing metastatic breast cancer as far as changes and concerns related to sexuality and intimacy were concerned, their efforts to cope with these concerns, information needs and intervention preferences.

A last contributing theme of this topic arose from the articles exploring and investigating *couple-based interventions*. Gorman et al. adapted a couple-based intervention to reduce reproductive and sexual distress by young and/or LGBTQ+ couples coping with breast or gynecologic cancer. The study of Fergus et al. aimed to evaluate the structure and content of an online psychological intervention for young couples facing breast cancer. The authors also examined the advantages and disadvantages of the program. The purpose of the systematic review of Hasdenteufel and Quintard was to propose an inventory of the experience of couples confronted with advanced cancer and to report the impact of psychosocial interventions focused on these dyads.

## Future directions

Several considerations emerge from this Research Topic, and they are critical to inform future studies. It is now clear that our scientific investigation should expand its current focus to include the experience of couples with different backgrounds, in terms of age, socio-economic level, ethnicity, culture, family background, sexual orientation, type of cancer, stage of cancer (Reese et al.; Fergus et al.; Lyons et al.; Saita et al.; Stulz et al.). Indeed, despite our best efforts, this issue presents mostly results from samples of heterosexual, white, high socio-cultural patients or couples with early stage breast or colon cancer. Future research should further investigate couples coping processes over time. Associations among individual-, partner-, couple- related factors with relational and health outcomes should be further considered (Fugmann et al.; Hasdenteufel and Quintard). Methodologically, future studies would also benefit from analyzing real-life interactions in order to increase ecological validity (Bodschwinna et al.; Rottmann et al.). Similarly, qualitative protocols would contribute to better understand each partner's representation of broader phenomena (e.g., end of life, expectations of partner, etc.) and therefore clarify how incongruence between partners' perception may influence their outcomes (Hasdenteufel and Quintard). In conclusion, these contributions all tend toward the same goal, namely to identify couples at greater risk and offer psychosocial care that is responsive to their needs and preferences (Fugmann et al.).

## Author contributions

All authors listed have made a substantial, direct, and intellectual contribution to the work and approved it for publication.
